# Structural Elucidation and Bioactivity of Biflavonoids from the Stems of *Wikstroemia taiwanensis*

**DOI:** 10.3390/ijms13011029

**Published:** 2012-01-18

**Authors:** Li-Yin Chen, Ih-Sheng Chen, Chien-Fang Peng

**Affiliations:** 1 Department of Pharmacy, Chia-Nan University of Pharmacy and Science, Tainan 71710, Taiwan; 2 School of Pharmacy, College of Pharmacy, Kaohsiung Medical University, Kaohsiung 80708, Taiwan; E-Mail: m635013@kmu.edu.tw; 3 Department of Medical Laboratory Science and Biotechnology, College of Health Sciences, Kaohsiung Medical University, Kaohsiung 80708, Taiwan; E-Mail: chfape@kmu.edu.tw

**Keywords:** *Wikstroemia taiwanensis*, Thymelaeaceae, stem, biflavonoid, antitubercular activity

## Abstract

Three new biflavonoids, wikstaiwanones A–C (**1**–**3**), along with four known compounds (**4**–**7**) were isolated from the stems of *Wikstroemia taiwanensis* (Thymelaeaceae). Their structures were elucidated by spectroscopic analysis. Compounds **4** and **5** showed antitubercular activity against *Mycobacterium tuberculosis* with MIC values of 15 μg/mL, respectively.

## 1. Introduction

**Figure 1:**
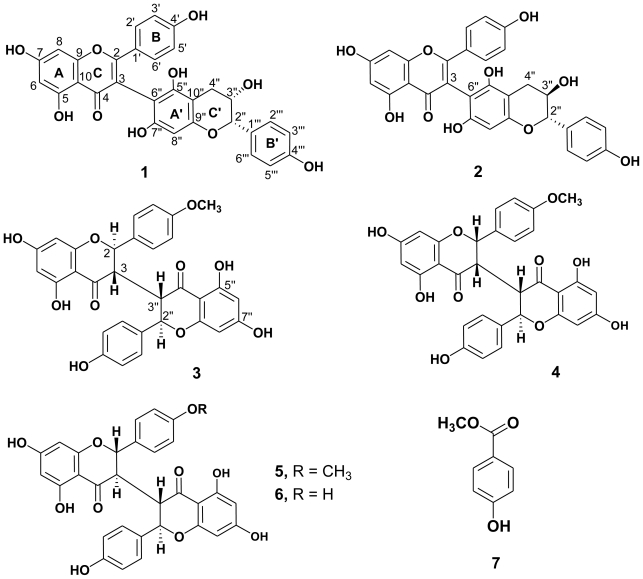
Structures of Compounds **1**–**7**.

Tuberculosis (TB), including multidrug resistant tuberculosis and extensively drug resistant tuberculosis, remains an epidemic in many regions of the world, with an estimated 8.8 million new cases and caused 1.1 million deaths globally in 2010 [1]. In Taiwan, it is also one of the most important communicable infectious diseases subject to official notification, with the highest number of confirmed cases and deaths [2]. Searching new sources of antitubercular agents from nature is crucial to curb the rise of drug-resistant strains. Examining an antitubercular activity from crude extracts of several Taiwan endemic plants, we found the methanolic extract of the stems of *Wikstroemia taiwanensis* showed potent activity against *Mycobacterium tuberculosis* H_37_Rv *in vitro W. taiwanensis* (Thymelaeaceae) is an endemic deciduous shrub growing in the south of Taiwan. Analysis of the chemical constituents and bioactivity of this plant has not been conducted previously. Bioactivity-directed fractionation of the active EtOAc layer of the stems of this species led to the isolation and characterization of three biflavonoids, wikstaiwanones A–C (**1**–**3**), along with three known biflavonoids, sikokianins B (**4**) [3], C (**5**) [4], isochamaejasmin (**6**) [5], and, methyl 4-hydroxybenzoate (**7**) [6] (Figure 1). This paper reports the structure elucidation of **1**–**3** and their antitubercular activities.

## 2. Results and Discussion

The present study focused on the fraction of the *W. taiwanensis* stem extract that displayed biological activity, which was confined to the EtOAc-soluble fraction. When this fraction was further separated by gel filtration into four parts, only one of them showed activity against *Mycobacterium tuberculosis* H_37_Rv. On separating this active part, compounds 1–7 were isolated, characterization of these compounds indicated 1–3 were new compounds.

### 2.1. Structure Elucidation of the New Isolates

**Figure 2:**
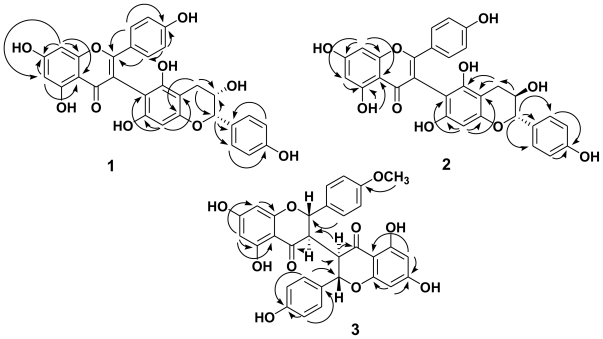
Key HMBC Correlations for **1**–**3**.

Compound **1** was isolated as an optically active yellowish amorphous powder, [α]_D_^25^−77.0 (*c* 0.03, MeOH). Its molecular formula was established as C_30_H_22_O_10_ by ESI-MS and HR-ESI-MS, with 20 degrees of unsaturation and a dimer composed of a flavone moiety and a flavanol moiety was proposed. The observed UV absorption peaks at 210, 217 sh, 226, 315 sh, and 335 sh nm in conjunction with a bathochromic shift observed after the addition of aq. KOH suggests the presence of a phenolic flavonoid moiety [7]. The IR spectrum showed absorption bands at 3450 cm^−1^ for a hydroxy group and at 1647 cm^−1^ for a carbonyl group. The ^1^H- and ^13^C-NMR spectra (Table 1) of **1** showed a trioxygenated flavone moiety [δ_C_ 160.7 (C-4′), 183.6 (C-4), 163.3 (C-5), and 166.0 (C-7)] with two *meta*-coupled protons [δ_H_ 6.18 (1H, d, *J* = 2.4 Hz, H-6), 6.32 (1H, d, *J* = 2.4 Hz, H-8)] in ring A and with a *para*-oxygenated phenyl group [δ_H_ 6.67 (2H, d, *J* = 9.0 Hz, H-3′, 5′), 7.26 (2H, d, *J* = 9.0 Hz, H-2′, 6′)] in ring C; and another 4‴, 3″, 5″, 7″-tetraoxygenated flavan moiety (δ_C_ 158.1, 68.9, 154.8 and 156.0) with a *para*-oxygenated phenyl group [δ_H_ 6.70 (2H, d, *J* = 9.0 Hz, H-3‴, 5‴), 7.10 (2H, d, *J* = 9.0 Hz, H-2‴, 6‴)] in ring C′ and an aromatic proton [δ_H_ 6.04 (1H, s), H-8″] in ring A′. The HMBC spectrum (Figure 2) showed correlations between the singlet H-8″ (δ_H_ 6.04) and C-6″ (δ_C_ 100.7) and the H-3 in ring C was lack in flavone moiety. These supported the connection between the flavone moiety and the flavan-3-ol moiety is via C-3 and C-6″ linkage. The molecular formula C_30_H_22_O_10_ supported the presence of seven hydroxy groups, which were confirmed by ^13^C-NMR, HMQC, NOESY and HMBC (Figure 2) experiments. The relative configurations of H-2″ and H-3″ were determined by NOESY spectrum, where H-2″ showed the correlation with H-3″. Comparing with the aglycone levorotatory optical activity of epiafzelechin 5-glucoside [α]_D_^25^ −63 (MeOH) [8], and the absolute configuration of **1** with [α]_D_^25^ −77.0 (*c* 0.03, MeOH) was suggested to be (2″*R*:3″*R*). It was found that when dissolved in different solvents, other compounds with (2″*R*:3″*R*) structure, e.g., (−)-epicatechin [9] and (−)-epigallocatechin [10] also displayed levorotatory optical activity [9,10]. Based on the evidence above, the structure of **1** was elucidated to be 5,7-dihydroxy-2-(4′-hydroxyphenyl)-3-(3″*R*,5″,7″-trihydroxy-2″*R*-(4‴-hydroxyphenyl)-3″,4″-dihydro-2*H*-chromen-6″-yl) -4*H*-chromen-4-one, and named wikstaiwanone A.

Compound **2** was obtained as pale yellowish amorphous powder with [α]_D_^25^ −23.3 (*c* 0.03, MeOH). Its molecular formula, C_30_H_22_O_10_, was same as **1** and established by ESI-MS and HR-ESI-MS. The IR, UV, and NMR (Table 1) spectra of **2** were similar to those of **1**, except that the different resonance of H-2″ (δ_H_ 4.53 (1H, d, *J* = 8.2 Hz), H-3″ (δ_H_ 3.68 (1H, br d, *J* = 8.2 Hz)) in **2** and H-2″ (δ_H_ 4.13 (1H, d, *J* = 7.8 Hz), H-3″ (δ_H_ 3.90 (1H, ddd, *J* = 8.4, 7.8, 6.0 Hz)) in **1**. This proposed **2** is a stereoisomer of **1**. Referring to the coupling constant *J* = 8.1 Hz of (−)-catechin with [α]_D_^25^ −14.9 (*c* 1.0, 50% aqueous acetone) [9], **2** was suggested to be the 3-epimer of **1** and H-2″/H-3″ were in *trans*-orientation. Thus the absolute configuration of **2** was determined as (2″*S*:3″*R*). Based on the above data, compound **2** was determined to be 5,7-dihydroxy-2-(4′-hydroxyphenyl)-3-(3″*R*,5″,7″-trihydroxy-2″*S*-(4‴-hydroxyphenyl)-3″,4″-dihydro-2*H*-chromen-6″-yl)-4*H*-chromen-4-one, namely wikstaiwanone B, which was further confirmed by HMQC, DEPT, NOESY, and HMBC (Figure 2) experiments.

Compound **3** was isolated as pale yellowish amorphous powder with [α]_D_^25^ +53.1 (*c* 0.058, MeOH). Its molecular formula was determined as C_31_H_24_O_10_ by ESI-MS and HR-ESI-MS with 15 unsaturated degrees. The ^1^H- and ^13^C-NMR spectra (Table 2) of **3** were similar to those of chamaejasmine [5] except a methoxy signal [δ_H_ 3.79 (3H, s, OCH_3_-4′), δ_C_ 159.6 (C-4′)] in **3** was in place of a hydroxy group at C-4′ in chamaejasmine. Thus the planar structure of **3** was determined as 3-(5,7-dihydroxy-2-(4-hydroxyphenyl)-4-oxochroman-3-yl)-5,7-dihydroxy-2-(4-methoxyphenyl)-2,3-dihydrochromen-4-one. The relation configurations at C-2/C-3 and C-3″/C-2″ positions were decided as *trans-trans* by comparison of *J* values of H-2/H-3 with 12.0 Hz and H-3″/H-2″ with 12.0 Hz, and no correlations between H-3/H-2, H-3″/H-2″ were observed. Additionally, H-3 and H-3′ (δ_H_ 2.60 (2H, dd, *J* = 18.0, 12.0 Hz)) with same chemical shift supported the H-3 and H-3′ being at same side. Referring to chamaejasmine [5] with [α]_D_ −61.2 (*c* 0.49, MeOH), compound **3** with dextrorotatory optical activity, [α]_D_ +53.1 (*c* 0.058, MeOH), indicated **3** is an enantiomer of chamaejasmine. According to the above evidence, the structure of **3** was elucidated as to be (2*R*,3*S*)-3-((2″*R*,3″*S*)-5″,7″-dihydroxy-2″-(4‴-hydroxyl-phenyl)-4-oxochroman-3-yl)-5,7-dihydroxy-2-(4-methoxyphenyl)-2,3-dihydrochromen-4-one, named wikstaiwanone C, which was further confirmed by HMQC, DEPT, NOESY and HMBC (Figure 2) techniques.

### 2.2. Antitubercular Activity

The amount of sample isolated for **1**–**7** ranged from about 3 to 42 mg, although all compounds underwent structural characterizations, only Shikokianins B (**4**) and C (**5**) (42.3 mg and 35.7 mg respectively) provided sufficient quantity for evaluating their *in vitro* antitubercular activity against *M. tuberculosis* H_37_Rv with the clinical used drug ethambutol (MIC 6.25 μg/mL) as a positive control. Results showed that both **4** and **5** have MIC values of about 15 μg/mL.

## 3. Experimental Section

### 3.1. General Experimental Procedures

Optical rotations were measured on a polarimeter (JASCO, Japan), UV spectra were obtained with a Jasco V-530 UV/VIS spectrophotometer. IR spectroscopic data were recorded on a Genesis II FTIR spectrophotometer with KBr pellets. NMR spectra were obtained on a Varian Unity Plus 400 spectrometer (400 MHz for ^1^H-NMR, 100 MHz for ^13^C-NMR) and Varian Unity Inova 600 spectrometer (600 MHz for ^1^H-NMR, 150 MHz for ^13^C-NMR). Chemical shifts were reported with respect to acetone*-d*_6_, methanol-*d*_4_ or DMSO solvents. Low-resolution MS spectra were obtained with Micromass Trio-2000 GC/MS, VG Biotech Quattro 5022, and JEOL-JMS-HX 100 mass spectrometers. The HRMS spectra were recorded on JEOL JMS-SX102A GC/LC/MS and Finnigan MAT-95XL high-resolution mass spectrometers. Silica gel (70–230 and 230–400 mesh; Merck) and Spherical C18 100 Å reversed phase silica gel (RP-18; particle size 20–40 μm; Silicycle) were used for column chromatography, and silica gel 60 F254 (Merck) and RP-18 F254S (Merck) were used for TLC and preparative TLC. Further purification was performed by HPLC (Hitachi; pump: L-2130; UV detector: L-2400).

### 3.2. Plant Material

Stems of *W. taiwanensis* were collected in May, 2008 at Mountain Goshifer, Pingtung County, and identified by Professor Ih-Sheng Chen. A voucher specimen (Chen 6163) has been deposited in the Herbarium of the College of Pharmacy, Kaohsiung Medical University, Kaohsiung, Taiwan.

### 3.3. Extraction and Isolation

Dried stems (9.8 kg) of *W. taiwanensis* were sliced to about 0.5cm in length and extracted three times, each time for 8 hours, with cold MeOH at room temperature to yield a MeOH extract, which was partitioned between EtOAc:H_2_O (1:1) to provide an EtOAc-soluble fraction (390 g). Both the aqueous and the EtOAc-soluble fractions were studies for their biological activities. The active EtOAc-soluble fraction (70 g) was subjected to Sephadex LH-20 column with CH_2_Cl_2_-MeOH (1:1) to obtained 4 fractions (1–4). The bioactive fraction 3 (24 g) was chromatographed on Silica gel CC and eluted with a CH_2_Cl_2_-MeOH-H_2_O gradient solvent system (50:3:1 to 6:4:1) to give fractions 3a-q. Fraction 3b (1.17 g) was subjected to a silica gel CC with CH_2_Cl_2_-EtOAc (15:1) to give 10 fractions (3b-1 to 3b-10). Fraction 3b-7 was subjected to MPLC and eluted with CH_2_Cl_2_-acetone (15:1) to give **5** (35.7 mg); Fraction 3b-8 was applied to MPLC eluting with CH_2_Cl_2_-MeOH (20:1) to give **6** (8.3 mg); and Fraction 3b-9 was purified with MPLC (RP_18_) eluting with MeOH-H_2_O (2:1) to give **4** (42.3 mg). Fraction 3e (2.56 g) was subjected to Sephadex LH-20 column and RP18 column successively to give **1** (3.8 mg), **2** (6 mg) and **7** (3 mg). Fraction 3f (708 mg) was eluted with MeOH–H_2_O (1:2) over RP_18_ column chromatography, purified with Sephadex LH-20 (MeOH) and ODS HPLC (254 nm, 250 × 10 mm, 70% CH_3_CN(aq.), 2.0 mL/min) to give **3** (12 mg).

*Wikstaiwanone A* (**1**): C_30_H_22_O_10_; Pale yellowish amorphous powder; [α]_D_^25^ = −77.0° (*c* = 0.03, MeOH); UV (MeOH): 210 (4.69), 217 sh (4.68), 266 (4.29), 315 sh (3.98), 355 sh (4.00); IR ν_max_ (KBr): 3450 (OH), 1647 (C=O) cm^−1^; ESI-MS, *m/z* 565 ([M+Na]^+^), HR-ESI-MS at *m*/*z* 565.1107 ([M+Na]^+^, C_30_H_22_O_10_Na, calc. 565.1111); ^1^H- and ^13^C-NMR data, see Table 1.

*Wikstaiwanone B* (**2**): C_30_H_22_O_10_; Pale yellowish amorphous powder; [α]_D_^24^ = −23.3° (*c =* 0.03, MeOH); UV (MeOH): 202 (4.72), 207 (4.72), 225 sh (4.51), 264 (4.36), 313 sh (4.04), 348 sh (3.94); IR ν_max_ (KBr): 3395 (OH), 1638 (C=O) cm^−1^; ESI-MS *m*/*z* 565 ([M+Na]^+^); HR-ESI-MS at *m*/*z* 565.1114 ([M+Na]^+^, C_30_H_22_O_10_Na, calc. 565.1111); ^1^H- and ^13^C-NMR data, see Table 1.

*Wikstaiwanone C* (**3**): C_31_H_24_O_10_; Pale yellowish amorphous powder; [α]_D_^25^ = +53.1° (*c* = 0.058, MeOH); UV (MeOH): 212 (4.61), 230 sh (4.47), 294 (4.55), 330 sh (3.77); IR ν_max_ (KBr): 3395 (OH), 1631, 1617 (C=O) cm^−1^; ESI-MS *m/z* 579 ([M+Na]^+^); HR-ESI-MS at *m*/*z* 579.1269 ([M+Na]^+^, C_31_H_24_O_10_Na, calc. 579.1267); ^1^H- and ^13^C-NMR data, see Table 2.

### 3.4. Antitubercular Activity Assay

The *in vitro* antitubercular activity of each tested compound was evaluated using the *Mycobacterium tuberculosis* H_37_Rv. *Middlebrook 7H10* agar was used to determine the minimum inhibitory concentration (MIC) values as recommended by the proportion method [22]. Briefly, each test compound was added to *Middlebrook 7H10* agar supplemented with oleic acid-albumin-dextrose-catalase (OADC) at 50–56° by serial dilution to yield a final concentrations of 100 to 0.8 μg/mL. Ten milliliter samples of each concentration of test compound-containing medium, was dispensed into plastic quadrant *Petri* dishes. Several colonies of the test isolate of *M. tuberculosis* were selected to make a suspension with *Middlebrook 7H10* broth, and used as the initial inoculum. The inoculum of test isolate of *M. tuberculosis* was prepared by diluting the initial inoculum in *Middlebrook 7H10* broth until turbidity was reduced to that equivalent to that of the *McFarland* No. 1 standard. Final suspensions were prepared by adding *Middlebrook 7H10* broth, and preparing 10^−2^ dilutions of the standardized bacterial suspensions. After solidification of the *Middlebrook 7H10* medium, 33 μL of the 10^−2^ dilution of the standardized bacterial suspensions was placed on each quadrant of the agar plates. The agar plates were then incubated at 35° with 10% CO_2_ for 2 weeks. The MIC is the lowest concentration of test compound that completely inhibited the growth of the test isolate of *M. tuberculosis*, as detected by the unaided eye.

## 4. Conclusions

Three new biflavonoids (**1**–**3**) together with four known compounds (**4**–**7**) were isolated from the stem of *W. taiwanensis* in this study. Several biflavonoids from Thymelaeaceae have been reported in a number of studies [11–18] and their biological activities evaluated [19–21]. Most of them contain C-3/C-3″ linkage with a symmetrical structure [11–14], except for wikstrols [4], genkwanols and stelleranol [14–17]. Compounds **1** and **2** were constituted by a flavone and a flavan-3-ol moieties and connected by an unusual C-3/C-6″ linkage, which was found for the first time in nature. The solubility in acetone of **1** and **2** is remarkably different, where **1** dissolved in acetone-*d_6_* slightly, **2** was readily soluble. Incidentally, their antitubercular properties have not previously been reported. In the study, compounds **4** and **5** were evaluated in this respect, and it was revealed that both have MIC values of 15 μg/mL, which is of the same order as the MIC value for ethambutol, a clinically used drug for tuberculosis, *i.e.* of 6.25 μg/mL. This indicated that the antitubercular activities of isolates from Thymelaeacea may be worth studying further.

## Figures and Tables

**Table 1:** ^1^H-NMR and ^13^C-NMR Data of Compounds **1** and **2**.

	1 a		2 b	
		
Position	δ_H_ (*J* in Hz)	δ_C_	δ_H_ (*J* in Hz)	δ_C_
2		165.0		164.5
3		114.2		114.4
4		183.6		183.4
5		163.3		164.2
6	6.18 (d, *J* = 2.4 Hz)	99.9	6.18 (d, *J* = 1.2 Hz)	99.9
7		166.0		165.3
8	6.32 (d, *J* = 2.4 Hz)	94.6	6.38 (d, *J* = 1.2 Hz)	94.7
9		159.5		159.3
10		105.0		105.7
1′		126.0		126.4
2′	7.26 (d, *J* = 9.0 Hz)	131.4	7.57 (d, *J* = 8.8 Hz)	131.9
3′	6.67 (d, *J* = 9.0 Hz)	115.6	6.87 (d, *J* = 8.8 Hz)	116.4
4′		160.7		161.1
5′	6.67 (d, *J* = 9.0 Hz)	115.6	6.87 (d, *J* = 8.8 Hz)	116.4
6′	7.26 (d, *J* = 9.0 Hz)	131.4	7.57 (d, *J* = 8.8 Hz)	131.9
2″	4.13 (d, *J* = 7.8 Hz)	82.8	4.53 (d, *J* = 8.2 Hz)	83.5
3″	3.90 (ddd, *J* = 7.8, 8.4, 6.0 Hz)	68.9	3.68 (br d, *J* = 8.2 Hz)	69.2
4″a	2.86 (dd, *J* = 6.0, 16.2 Hz)	29.1	2.91 (dd, *J* = 16.0, 5.6 Hz)	30.3
4″b	2.46 (dd, *J* = 8.4, 16.2 Hz)		2.56 (dd, *J* = 16.0, 6.8 Hz)	
5″		154.8		155.0
6″		100.7		101.0
7″		156.0		156.7
8″	6.04 (s)	96.5	6.15 (s)	97.0
9″		157.8		157.7
10″		101.5		101.6
1‴		131.6		131.9
2‴	7.10 (d, *J* = 9.0 Hz)	129.5	6.87 (d, *J* = 8.8 Hz)	130.1
3‴	6.70 (d, *J* = 9.0 Hz)	115.8	6.65 (d, *J* = 8.8 Hz)	116.2
4‴		158.1		158.5
5‴	6.70 (d, *J* = 9.0 Hz)	115.8	6.65 (d, *J* = 8.8 Hz)	116.2
6‴	7.10 (d, *J* = 9.0 Hz)	129.5	6.87 (d, *J* = 8.8 Hz)	130.1

a ^1^H (600 MHz, CD_3_OD), ^13^C (150 MHz, CD_3_OD);

b ^1^H (400 MHz, acetone-*d*_6_), ^13^C (100 MHz, acetone-*d*_6_).

**Table 2:** ^1^H-NMR, ^13^C-NMR, and HMBC Data of Compound **3**.

	3 a		
	
Position	δ_H_ (*J* in Hz)	δ_C_	HMBC
2	5.70 (d, *J* = 12.0 Hz)	82.3	H-3, H-3″, H-2′, H-6′
3	2.60 (dd, *J* = 12.0, 18.0 Hz)	48.5	H-3″
4		193.0	H-3
5		161.8	H-6
6	5.46 (br d)	96.7	H-8
7		161.9	H-8
8	5.54 (s)	97.5	H-6
9		163.5	H-8
10		99.0	H-6, H-8
1′		128.9	H-2, H-2′, H-3′, H-5′, H-6′
2′	6.95 (d, *J* = 8.0 Hz)	129.1	H-2, H-3′, H-6′
3′	6.87 (d, *J* = 8.0 Hz)	113.8	H-2′, H-5′, H-6′
4′		159.6	H-2, H-3′, H-5′, H-6′, Ome
5′	6.87 (d, *J* = 8.0 Hz)	113.8	H-2′, H-3, H-6′
6′	6.95 (d, *J* = 8.0 Hz)	129.1	H-2, H-2′, H-5′
2′	5.64 (d, *J* = 12.0 Hz)	82.5	H-3, H-3″, H-2‴, H-6‴
3′	2.60 (dd, *J* = 12.0, 18.0 Hz)	48.6	H-3
4″		193.0	H-3″
5″		161.8	H-6″
6″	5.46 (br d)	96.6	H-8″
7″		161.9	H-8″
8″	5.54 (s)	97.6	H-6″
9″		163.5	H-8″
10″		99.1	H-6″, H-8″
1‴		127.1	H-2″, H-3‴, H-5‴
2‴	6.78 (d, *J* = 8.0 Hz)	128.9	H-2″, H-3‴, H-6‴
3‴	6.70 (d, *J* = 8.0 Hz)	115.3	H-2‴, H-5‴
4‴		158.0	H-2‴, H-3‴, H-5‴, H-6‴
5‴	6.70 (d, *J* = 8.0 Hz)	115.3	H-3‴, H-6‴
6‴	6.78 (d, *J* = 8.0 Hz)	128.9	H-2″, H-2‴, H-5‴
OMe	3.79 (s)	55.1	

a ^1^H (600 MHz, DMSO-*d*_6_), ^13^C (150 MHz, DMSO-*d*_6_).
